# Time to early initiation of postnatal care service utilization and its predictors among women who gave births in the last 2 years in Ethiopia: a shared frailty model

**DOI:** 10.1186/s13690-021-00575-7

**Published:** 2021-04-15

**Authors:** Ayal Debie, Getayeneh Antehunegn Tesema

**Affiliations:** 1 Department of Health Systems and Policy, Institute of Public Health, University of Gondar, P.O. Box: 196, Gondar, Ethiopia; 2 Department of Epidemiology and Biostatistics, Institute of Public Health, University of Gondar, Gondar, Ethiopia

**Keywords:** Time to early initiation, Postnatal care, Predictors, Ethiopia

## Abstract

**Background:**

Most maternal and infant deaths occurred within the first month after birth. Nearly half of the maternal deaths and more than a million newborn deaths occurred within the first day of life but these were preventable through early initiation of postnatal care (PNC) services. However, the available evidence on the level of early initiation of PNC service utilization was not adequate to inform policy decisions. Therefore, this study aimed to assess time to early initiation of postnatal care and its predictors using the 2016 Ethiopian Demography and Health Survey (EDHS) datasets.

**Methods:**

Two-stage stratified cluster sampling technique by separating each region into urban and rural areas. A total weighted sample of 6364 women of the 2016 EDHS datasets who gave birth within 2 years preceding the survey was used. Time to early initiation of the PNC visit was estimated using the Kaplan-Meier (K-M) method. Shared frailty model with baseline distributions (Weibull, Gompertz, exponential, log-logistic, and lognormal) and frailty distributions (gamma and inverse Gaussian) were used by taking enumeration areas/clusters as a random effect for predictors of time to early initiation of PNC visit. The adjusted hazard ratio (AHR) with a 95% confidence interval (CI) and *p*-value less than 0.05 were used to declare the significant predictor variables for time to early initiation of the PNC service utilization.

**Results:**

The prevalence of women who utilized PNC services within 42 days was 13.27% (95% CI, 12.46, 14.13). Among these women, only 1.73% of them had got within the first 24 h of birth; 4.66% of them received within 48–72 h and 1.74% of them also had got within 7–14 days. Variables, such as parity (AHR = 1.61, 95% CI: 1.21, 2.15), media exposure (AHR = 1.42, 95% CI: 1.21, 1.68), place of delivery (AHR = 14.36, 95% CI: 11.76, 17.53), caesarean delivery (AHR = 2.17, 95% CI: 1.60, 2.95) and antenatal care visit (AHR = 2.07, 95% CI: 1.63, 2.63) had the higher hazard for PNC services utilization. On the other hand, women who faced with healthcare access problems (AHR = 0.74, 95% CI: 0.60, 0.87) had a lower hazard of PNC service utilization.

**Conclusion:**

The overall postnatal care service utilization among women in the survey was low, particularly within the first 24 h of delivery. Policy-makers and implementers should promote the utilization of antenatal care and institutional delivery using mass media to increase the continuum of maternity care. The government should also design a new approach to enhance the uptake of postnatal care services for poor households and to scale up the PNC services, including the different possibilities for women who give births at the health facilities and homes. Future researchers had better assess the capacity and accessibility of the local health systems, the level of decentralized decision making, common cultural practices, knowledge, attitude, and perception of mothers towards PNC service utilization.

**Supplementary Information:**

The online version contains supplementary material available at 10.1186/s13690-021-00575-7.

## Background

Postnatal care is care given to the mother and her newborn baby immediately after the birth of the placenta and the first 42 days of life. The postnatal period is the days and weeks following childbirth and it is a critical phase in the lives of mothers and newborn babies. All mothers and babies need at least four postnatal checkups in the first 6 weeks. These visits are on the first day (24 h), day 3 (48–72 h), between days 7–14, and 6 weeks [1]. Almost half of the postnatal maternal deaths occur within the first 24 h of birth, and 66% occur during the first week of birth [2]. In 2013, about 2.8 million newborns died in their first month of life and 1 million of these died on the first day of birth. The majority of maternal and neonatal deaths occur during childbirth and the postnatal period and approximately 303,000 maternal deaths occurred globally in 2015 [3].

To curb this problem, the new globally adopted agenda between 2016 and 2030 as a part of the sustainable development goal aimed to reduce the maternal mortality ratio to 70 per 100,000 live births by addressing all maternal health care services for every woman as a top priority [4]. A substantial number of women had an antenatal visit before getting other subsequent maternal health care services in many countries, however, they failed to access skilled birth attendants and postnatal care [5]. Only 67 and 48% of women gave birth with the assistance of skilled personnel in South-East Asia and sub-Saharan Africa, respectively [6]. Below half of the women received a postnatal care visit within the first 2 days of childbirth. The findings from the 23 sub-Saharan African countries indicated that only 13% of women who delivered at home received postnatal care within the first 2 days of birth [7].

Ethiopia is organized in the nine national regional states and the two city administrations. The nine regions are Tigray, Afar, Amhara, Benishangul-Gumuz, Gambela, Harari, Oromia, Somali, and Southern Nations, Nationalities, and People’s of Region (SNNP) regions, and the two city administrations are Addis Ababa and Dire-Dawa. Each region and city administrations have its own autonomous Regional Health Bureau (RHB). The healthcare delivery in Ethiopia is organized in a three-tier System. The first, at the district level, is the Primary Healthcare Unit (PHCU). The PHCU comprises one primary hospital, four health, and five health posts that are attached to each health center. The second level comprises general hospitals, while the third level comprises specialized hospitals.

In the Ethiopian healthcare system, some public health services have been provided to all citizens free of charge regardless of the level of income. This has occurred because of the nature of these activities and the need to promote the use of certain health care services. These services are tuberculosis treatment and follow-up, HIV/AIDS testing and treatment, immunization service, maternal health care services, including antenatal care, delivery care, postnatal care, and family planning service.

Ethiopia is one of the countries with low coverage of both access to basic health services and health service utilization, a high geographical (urban-rural area) disparity as well as a high catastrophic out-of-pocket spending for healthcare services. However, exemption services or cost-sharing for high-priority interventions are the key strategies of the government of Ethiopia to achieve Universal Health Coverage (UHC) [8, 9]. The PNC services are made accessible to the village level with lower or no cost in Ethiopia but only 17% of women received a postnatal check-up in the EDHS 2016 which is far from 26% of institutional delivery service utilization [10]. The PNC service utilization in Debre Markos town and Dembecha district in northwest Ethiopia was 33.5 and 34.8%, respectively [11, 12]. Moreover, PNC service utilization among women in the Jimma zone was 58.5 and 36.7% in Loma district [13], Ethiopia. Even though the trends of reproductive health service indicators had significant improvement, the gap in the continuum of maternal healthcare services remains remarkably high. This study, therefore, aimed to analyze the time to early initiation of postnatal care and its predictors among women who gave birth in the last 2 years in Ethiopia using the 2016 EDHS datasets.

## Methods

### Data source and sampling procedure

**Fig. 1:**
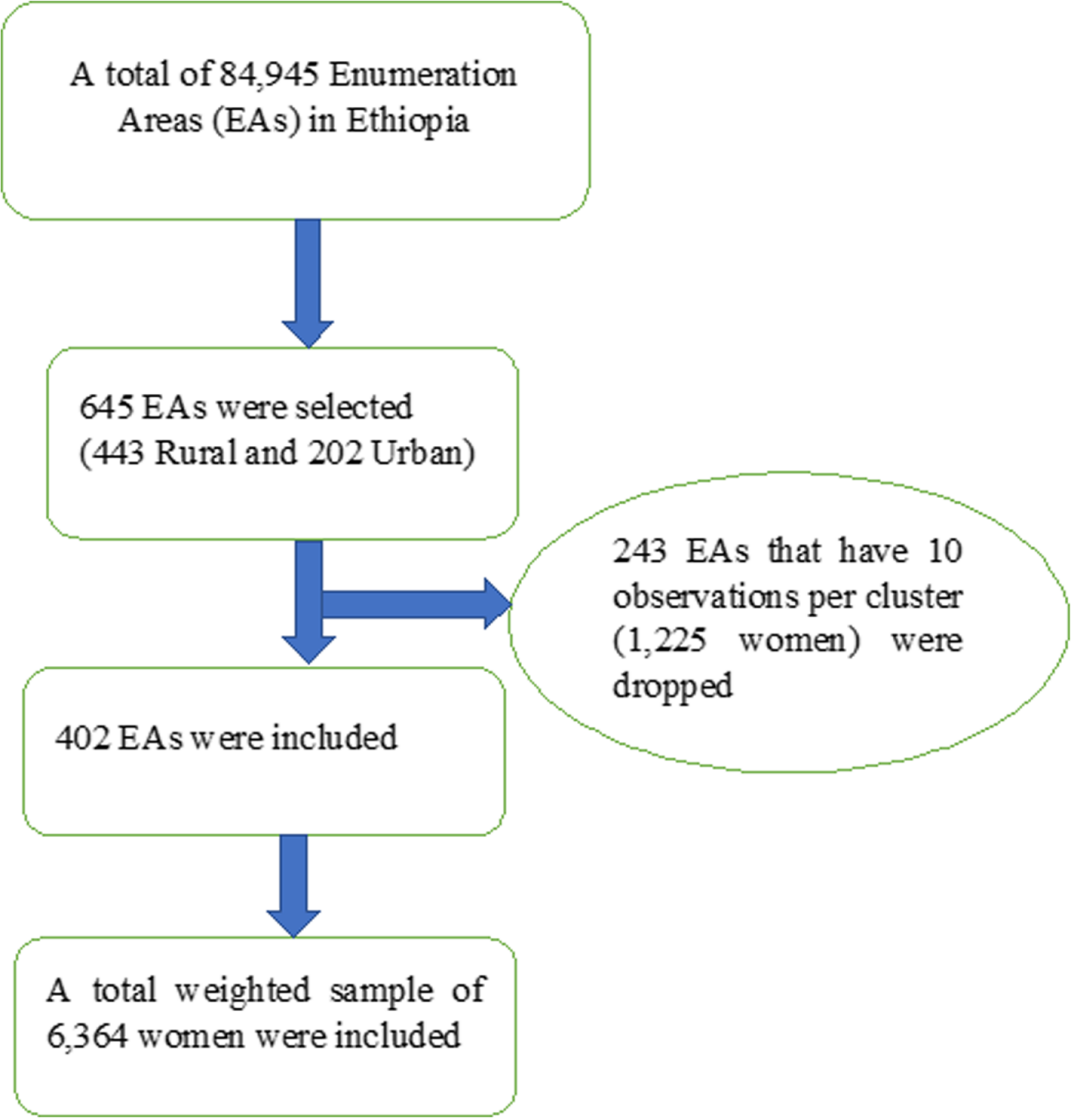
The sampling procedures for selecting the study participants in EDHS 2016

Secondary data analysis was conducted based on the EDHS 2016. The EDHS used a stratified two-stage cluster sampling technique selected using the 2007 Population and Housing Census as a sampling frame. A total of 84,915 Enumeration Areas (EAs) were created in Ethiopia and stratification was done by dividing each of the nine regions into urban and rural areas and a total of 21 sampling strata were formed. In the first stage, 645 enumeration areas (202 in the urban area) were selected with proportional allocation to the size of the enumeration areas (EAs) with an independent selection of each sampling stratum. A total of 243 EAs that have less than 10 observations per cluster (a total of 1225 observations) were dropped. A total of 402 EAs were, therefore, included for analysis to get a reliable estimate. A minimum of 10 and maximum of 21 women or on average 15 women per EAs were selected using systematic sampling technique. A total weighted sample of 6364 women who gave birth within 2 years preceding the survey were included for this study (Fig. 1). The detailed sampling procedure was presented in the full EDHS 2016 report [14].

### Study variables

Women who gave birth within 2 years preceding the survey were considered in this study and those who had PNC visits within 42 days of birth were considered as a success while those who didn’t have a visit were treated as a failure. It is defined as the time of first PNC checkup within the first 42 days of birth. The time to first PNC visit was recorded in days if the women have a PNC visit within 42 days of birth. The event is binary form, coded as “1” if a woman had a PNC visit within 42 days of birth and “0” if the women didn’t have a PNC visit within the 42 days. The independent variables considered for this study were categorized as socio-demographic and economic variables (residence, region, religion, maternal education, husband education, maternal occupation, sex of household head, distance to the health facility, and wealth status), and obstetric related factors (the type of gestation, preceding birth interval, ANC visit, place of delivery, mode of delivery, parity, birth order).

### Data management and analysis

The data were weighted using sampling weight, primary sampling unit, and strata before any statistical analysis to restore the representativeness of the survey and to take into account the sampling design to get reliable statistical estimates. STATA version 14 software was used for the descriptive as well as for the frailty analysis. Because of the hierarchical structure of EDHS data, women are nested within a cluster and we expect that women within the same cluster may be more similar to each other than women in the rest of the country. This violates the assumption of the traditional regression model which is the independence of observations and equal variance across clusters. A total of 243 EAs that have less than 10 observations per cluster (a total of 1225 observations) were dropped from the analysis to balance the size of clusters and detect the random effect efficiently. The standard survival analysis models are applicable when the time to event data is independent but the EDHS was a cluster survey that has hierarchical nature and assumed to be correlated at the cluster level. The correlation could be due to unobserved cluster or EAs specific covariates and assumes that time to early initiation of PNC service of a mother is a function of measured variables and a random (frailty) on the baseline hazard to the unobserved cluster effect. Schoenfeld residual global test was applied to check the Proportional Hazard (PH) assumption, and it was violated with a *p*-value < 0.05 (Supplementary file 1).

Parametric survival models were fitted since the PH assumption was violated. The EDHS data has a hierarchical structure and a frailty model (random effect survival model) was used to check whether there is clustering or not. The theta was significant at the null model (θ = 1.47, 95% CI: 1.26, 1.72). It indicates that there was unobserved heterogeneity or shared frailty in which women in one cluster were more likely to be correlated with women in the same cluster. Shared frailty model with baseline distributions (Weibull, Gompertz, Exponential, log-logistic, and lognormal) and frailty distributions (gamma and inverse Gaussian) were used by taking EAs of v001 as a random effect for predictors of time to early initiation of PNC visit among women who gave births. A Weibull gamma shared frailty model was the best-fitted model for this data since it has the smallest deviance and AIC values. Variables with a *p*-value less 0.20 in the uni-variable of the Weibull gamma shared frailty analysis were included in the multivariable analysis. The Hazard Ratio (HR) with 95% Confidence Interval (CI) and *p*-value less than 0.05 were used to declare the significant predictor variables for time to early initiation of the PNC service utilization.

The model was formulated as:

$$ hij\left(t\vert xij,\mathrm{ui}\right)=\mathrm{uih}0\left(\mathrm{t}\right)\ {\mathrm{e}}^{\beta\prime } xij\Big) $$

For time to event data, where i (1 ………., n) denotes the cluster, while j (1, ……, n) denotes the subjects (woman) within the cluster. The frailty, *u i* is a random positive quantity shared within groups, whereas hij (t│xij,ui) is the probability of women initiating PNC service at time t; h0(t) is the baseline hazard and Xij is a vector of covariates with the associated vector of fixed parameters β. Time to early initiation of the PNC visit was estimated using the Kaplan-Meier (K-M) method. The log-rank test was used to compare survival time between groups of categorical variables.

## Results

### Socio-demographic and economic characteristics of women

A total of 6364 women who gave birth within 2 years the survey was included in the study. Of these, 2825 (44.4%) were in the Oromia region, and 6033 (94.8%) were rural residents. Half (49.7%) of the women were in the age groups of 25–34 years. About 4267 (67.1%) of the women and 2967 (46.6%) of their husbands had no formal education **(**Table 1**)**.

**Table 1:** Socio- economic characteristics of women who gave birth in the last 2 years in Ethiopia, 2016

Variables	Weighted frequency (%)
**Region**	
Tigray	487 (7.7)
Afar	64 (1.0)
Amhara	1149 (18.1)
Oromia	2825 (44.4)
Somalia	234 (3.7)
Benishangul-Gumuz	65 (1.0)
SNNPRs	1431 (22.5)
Gambella	14 (0.2)
Harari	11 (0.2)
Addis Ababa	64 (1.0)
Dire Dawa	20 (0.3)
**Residence**	
Rural	6033 (94.8)
Urban	331 (5.2)
**Maternal age**	
15–24	1585 (24.9)
25–34	3163 (49.7)
≥ 35	1616 (25.4)
**Religion**	
Orthodox	2196 (34.5)
Muslim	2492 (39.2)
Protestant	1461 (23.0)
Others	215 (3.4)
**Maternal education**	
No	4267 (67.1)
Primary	1801 (28.3)
Secondary and/or higher	296 (4.6)
**Husband education**	
No	2967 (46.6)
Primary	2393 (37.6)
Secondary and/or higher	1004 (15.8)
**Wealth status**	
Poorest	1543 (24.3)
Poorer	1548 (24.3)
Middle	1441 (22.6)
Richer	1216 (19.1)
Richest	616 (9.7)
**Media exposure**	
No	4453 (70)
Yes	1911 (30)
**Sex of household head**	
Male	5488 (86.2)
Female	876 (13.8)

### Obstetric related characteristics of women

A total of 2595 (40.8%) mothers had 2–4 births and 4745 (75.6%) gave births at home among 6364 women who gave birth in the last 2 years. The majority (59%) of women had ANC visits during pregnancy and only 1.2% gave birth through caesarean section. About 5366 (84.3%) of the women had a preceding birth interval of 2 or more years, and 95 (1.5%) of mothers had twin birth **(**Table 2**)**.

**Table 2:** Obstetric characteristics of women who gave birth in the last 2 years in Ethiopia, 2016 (*n* = 6364)

Variables	Weighted frequency (%)
**Parity**	
1	1097 (17.2)
2–4	2595 (40.8)
≥ 5	2672 (42.0)
**Outcome of birth**	
Alive	5818 (91.4)
Stillbirth	546 (8.6)
**Place of delivery**	
Home	4745 (75.6)
Health institution	1618 (25.4)
**Mode of delivery**	
Spontaneous vaginal delivery (SVD)	6286 (98.8)
Caesarean delivery	77 (1.2)
**ANC visit during pregnancy**	
No	2606 (41.0)
Yes	3758 (59.0)
**Sex of child**	
Male	3308 (52.0)
Female	3056 (48.0)
**Preceding birth interval in years**	
< 2	998 (15.7)
≥ 2	5366 (84.3)
**Number of births**	
Single	6269 (98.5)
Multiple	95 (1.5)
**Health care access problem**	
A big problem	4038 (63.5)
Not a big problem	2326 (36.5)
**Health care decision making autonomy**	
Respondent alone	768 (12.1)
Jointly with husband	3929 (61.7)
Husband or parent only	1667 (26.2)

### Predictors of time to early initiation of PNC

**Fig. 2:**
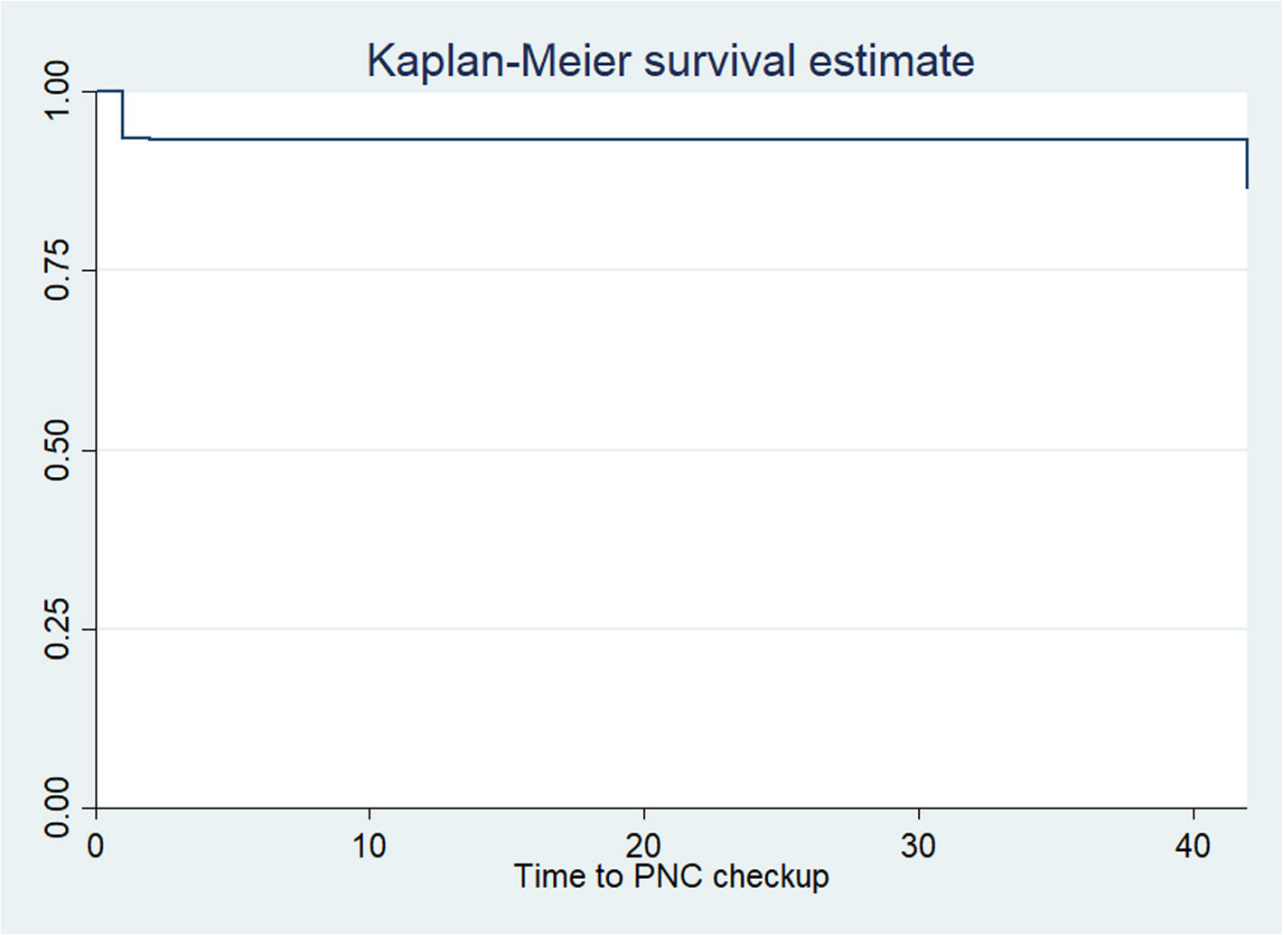
Overall Kaplan-Meier failure curve for the probability of PNC visits among mothers in Ethiopia, 2016

The overall prevalence of women who had utilized postnatal care services within 42 days was 13.27% (95% CI: 12.46, 14.13). Among these women, only 1.73% of them had got within the first 24 h of birth and 4.66% of them received within 48–72 h. On top of that, 1.74% of women received their first PNC from 7 to 14 days of the postpartum period. Kaplan Meier’s failure curve was used to compare PNC service utilization within 42 days among categories of independent variables graphically. The Kaplan Meier(K-M) failure curve was done for all possible predictors. The K-M curve differs for various educational categories of women, ANC visit, and place of delivery, birth outcome, and mode of delivery. The overall Kaplan-Meier failure curve indicated that the probability of PNC visits was highest on the first-day postpartum period that is relatively increased as follow-up time increases **(**Fig. 2**)**.

### Log-rank test for the predictors of PNC

Residence, ANC, birth outcome, place of delivery, preceding birth interval, region, maternal education, husband education, respondent age, twin pregnancy, parity, and wealth index showed a statistically significant difference in probability of early initiation of PNC visit with the log-rank test of *p* < 0.05 (Supplementary file 2).

#### Model comparison and diagnostics for PNC

The theta was significant at the null model (θ = 16, 95% CI: 0.10, 0.29), LR test of theta = 0: chibar2(01) = 22.60, Prob > = chibar2 = 0.0001. The shared frailty model with the Weibull baseline hazard function with gamma frailty had the smallest AIC, BIC, deviance, and theta value than the other models and was the best-fitted model for identifying predictors of time to early initiation of PNC service (Supplementary file 3).

### Multivariable survival analysis

In the multivariable gamma shared Weibull model; type of birth, parity, preceding birth interval, media exposure, health care access problem, place of delivery, ANC visit, and mode of delivery were significant predictors of early initiation of PNC checkup. Women who had twin births 2.05 times (AHR = 2.05, 95% CI: 1.32, 3.18) more likely to utilize PNC service than singletons. Mothers who had 2–4 births and 5 or more births were 1.52 times (AHR = 1.52, 95% CI: 1.23, 1.87) and 1.61 times (AHR = 1.61, 95% CI: 1.21, 2.15) higher likelihood of PNC service utilization compared with their counterparts, correspondingly. Mothers who had media exposure (AHR = 1.42, 95% CI: 1.21, 1.68), institutional delivery (AHR = 14.36, 95% CI: 11.76, 17.53), and caesarean delivery (AHR = 2.17, 95% CI: 1.60, 2.95) had the higher hazard of PNC service utilization compared with their counterparts, respectively. Similarly, women who made ANC visit had two folds (AHR = 2.07, 95% CI: 1.63, 2.3) higher likelihood of PNC service utilization compared with those who had no ANC attendance. On the contrary, women who faced big healthcare access problems (AHR = 0.74, 95% CI: 0.63, 0.87) decreases the hazard of PNC service utilization by 26% **(**Table 3**)**.

**Table 3:** Weibull gamma shared frailty model for the predictors of early initiation of PNC service utilization in Ethiopia, 2016

Variables	PNC		Crude Hazard Ratio (CHR) with 95% CI	Adjusted Hazard Ratio (AHR) with 95% CI
	No	Yes		
**Residence**				
Urban	196	135	1	1
Rural	5323	710	0.23 [0.13, 0.35]	1.06 [0.76, 1.46]
**Maternal age**				
15–24	1332	253	1	1
25–34	2738	425	0.83 [0.70, 0.97]	0.98 [0.81, 1.20]
≥ 35	1450	166	0.66 [0.54, 0.81]	0.87 [0.65, 1.17]
**Maternal education status**				
No	3863	403	1	1
Primary	1482	319	1.77 [1.51, 2.07]	1.04 [0.88, 1.25]
Secondary or higher	174	122	3.62 [2.85, 4.60]	1.04 [0.79, 1.39]
**Wealth status**				
Poorest	1429	114	1	1
Poorer	1382	166	1.34 [1.04, 1.74]	0.96 [0.75, 1.24]
Middle	1245	197	1.65 [1.28, 2.14]	1.17 [0.75, 1.56]
Richer	1055	160	1.64 [1.25, 2.16]	0.91 [0.69, 1.19]
Richest	408	208	3.56 [2.68, 4.73]	1.20 [0.88, 1.65]
**Sex of household**				
Male	5448	1025	1	1
Female	866	250	1.23 [1.06, 1.43]	1.02 [0.83, 1.25]
**Type of birth**				
Single	6235	1235	1	1
Twin	78	41	2.21 [1.58, 3.09]	2.05 [1.32, 3.18] *
**Parity**				
1	887	210	1	1
2–4	2220	374	0.79 [0.66, 0.95]	1.52 [1.23, 1.87]*
≥ 5	2412	261	0.59 [0.49, 0.72]	1.61 [1.21, 2.15]*
**Birth interval**				
< 2 years	919	79	1	1
≥ 2 years	4600	765	1.69 [1.33, 2.15]	1.35 [1.06, 1.73] ^*^
**Husband education**				
No	2674	293	1	1
Primary	2077	316	1.21 [1.02, 1.44]	0.89 [0.75, 1.06]
Secondary and above	768	236	2.01 [1.66, 2.44]	1.17 [0.93, 1.47]
**Media exposure**				
No	4026	428	1	1
Yes	1494	417	1.96 [1.68, 2.28]	1.42 [1.21, 1.68]*
**Healthcare access**				
No big problem	1083	428	1	1
A big problem	4436	417	0.54 [0.47, 0.62]	0.74 [0.63, 0.87] ^*^
**Women’s decision making autonomy**				
Respondent alone	640	128	1.19 [1.01, 1.42]	0.97 [0.75, 1.06]
Jointly with partner	3404	525	1.12 [0.88, 1.42]	0.97 [0.76, 1.24]
Partner only	1476	191	1	1
**Birth outcome**				
Alive	5056	762	1	1
Stillbirth	463	82	1.14 [0.90, 1.44]	1.09 [0.86, 1.39]
**Place of delivery**				
Home	4597	148	1	1
Health facility	922	696	19.66 [16.33, 23.68]	14.36 [11.76, 17.53] *
**Mode of delivery**				
SVD	5497	789	1	1
Caesarean delivery	22	56	6.15 [4.47, 8.47]	2.17 [1.60, 2.95] *
**ANC visit**				
No	2515	91	1	1
Yes	3004	753	4.81 [3.82, 6.05]	2.07 [1.63, 2.63] *

## Discussion

Despite the significant improvement in maternal and child healthcare interventions, maternal mortality ratio (MMR) was 412 per 100,000 live births and the neonatal mortality rate was 29 per 1000 livebirths in Ethiopia [10]. These are still our challenges to achieve the HSTP goals of MMR of 199 per 100,000 live births and neonatal mortality of 10 per 1000 live births in the country [15]. Most neonatal deaths usually occur in the first 24 h of life, and three -quarters of neonatal deaths occur in the first week after birth [16]. Provide PNC in the first 24 h to all mothers and babies, regardless of where the birth occurs is desirable [17]. The movement of mothers and babies is culturally restricted for a period of seclusion after delivery, particularly in African communities. Keeping mothers and babies indoors for the first month after birth, and seeking formal health care is often delayed when mothers or babies become ill during the period of seclusion [18, 19].

This study has investigated the association of socioeconomic, demographic, and environmental.

factors with the likelihood of PNC service utilization used by accounting for gamma-distributed shared frailties at the cluster-level. In this study, about 13.27% of mothers received postnatal check-ups from a trained health service provider within 42 days of delivery. This result was higher than in Abi Adi town, Ethiopia (11.90%) [20]. On the contrary, the finding was lower than the studies conducted in Jabitena, Ethiopia (20.20%) [21], North Shoa (928.4%) [22], Ambo (33.4%) [23], Assela (72.7%) [24], Jimma (58.5%) [25], Debre Markos (33.5%) [11], Dembecha (34.8%) [12], Loma (36.7%) [13], Lemo (51.4%) [26], Ethiopia, South Africa (59.6%) [27], North Eastern Nigeria (16.9%) [28], Bangladesh (55.6%) [29], Myanmar(25.2%) [30] and Nepal (34%) [31]. Moreover, only 1.73 and 6.39% of mothers received PNC services within the first 24 and 72 h of the postpartum period in the survey. This was lower than the studies done in Bangladesh (27.3%) [29] and Nepal (19%) [31]. The possible justification for this variation might be due to the differences in study settings, areas, participant characteristics, design, and period. This could be some of the previous studies might be done only among urban women who attended health facilities since these participants might have a higher chance to access media exposure and maternal healthcare services.

The hazard of PNC service utilization was higher for women who had media exposure than media non-users. This finding was supported by the studies done in India [32] and Nepal [31]. This might be due to those women who had media exposure could have adequate information about maternal health care services. Women who had twin births and a greater number of parities had a higher hazard of PNC service utilization compared with their counterparts. This could be because mothers who had twin births and more parities are tending to have greater perceived susceptibility to a wide range of complications therefore frequent visits to the health institution would be the strategy to minimize their perceived risks. On the contrary, this finding contradicted studies conducted in North Shoa, Ethiopia, and Nepal [31]. This might be justified by the fact that first-time mothers are usually dependent upon the support of health professionals and their families for infant care and feeding practices. Besides, they would be very curious about the health of their newborn baby, thus, these needs might be satisfied through frequent contact with health facilities.

Mothers who had ANC follow up had a higher hazard for PNC service utilization. This result was in line with the studies conducted in Ambo [23], Assela [24], Jimma [25], Dembecha [12], Lemo [26], Ethiopia, South Africa [27], and Nepal [31]. The possible explanation for the strong positive association between ANC attendance and PNC service utilization might be that mothers and their families receive health education and advise during ANC visits and thus become access to learn about the benefits of PNC services follow up in health care facilities.

The hazard of PNC service was higher among health facility delivery attended women compared with home-delivered mothers. The finding was in line with the studies done in Abi Adi town, Ethiopia [20], Gulele Sub-city, Addis Ababa [33], Jimma [25], Debre Markos [11], Jabitena [21], Gondar Zuria [34], Loma [13], Lemo [26], Ethiopia, Royal king of Cambodia [35], Bangladesh [36], and South Africa [27], which indicates that giving birth at health institution has significantly associated with PNC service utilization. The possible explanation could be attributed to the fact that women who gave their last birth in health institution have a greater opportunity for health education related to PNC services at the time of delivery and thus get access to learning about the types, benefits, and availabilities of PNC services during their stay in the health institutions. This exposure increases healthcare-seeking behavior to prevent maternal and neonatal complications compared to those mothers who gave birth at home. Furthermore, those women who gave birth at home belong to a more traditional cohort and thus become less likely to use postnatal care services [37].

Women who gave birth by caesarean section had a higher hazard of postnatal care service utilization compared with women who gave birth via spontaneous vaginal delivery. This finding was supported by the studies done in North Shoa [22], Debre Markos [11], Ethiopia, and Tanzania [38]. The possible justification could be mothers who had operative delivery are tending to have greater perceived susceptibility to a wide range of postoperative complications therefore frequent return to the health institution would be the strategy to minimize their perceived risks.

On the contrary, women who faced healthcare access problems had a lower hazard of PNC service utilization than mothers who did not face healthcare access problems. This could be because the availability of services and understanding of the importance of postnatal care is vital to improving its uptake, however, physical distance limits women’s willingness and ability to seek health care, particularly when appropriate transportation is scarce and communications and the terrain are difficult [39].

This finding indicated that the PNC services are among the weakest of all reproductive and child healthcare services in Ethiopia. This might alarm the policy on how can they increase the coverage of early initiation of PNC services to provide integrated maternal and newborn care in the postnatal period. This can also give a chance for policymakers to decide and integrate the PNC services with the existing strategies and programs, especially in childbirth care, Integrated Management of Childhood Illness (IMCI), nutrition promotion, prevention of mother-to-child transmission of HIV, and immunization. This might also give a hint to evaluate the approaches used to promote maternal healthcare services that have been delivered using the community health workers (CHWs), mother support group (MSG), women health development army (HAD), and the use of mass media for maternal services.

The Weibull gamma shared frailty model to identify the significant predictors for the early initiation of PNC visits. This model was chosen since the DHS data has hierarchical nature and could violate the basic assumptions of standard survival models. Frailty model is therefore important to get a reliable estimate and standard error since it takes into account the clustering effect while estimating the coefficients and standard errors by introducing the EAs as a random effect. The standard survival model could not give a reliable estimate and standard error for such data. A comparison of the survival models was done using deviance, AIC, and theta values to decide which model is appropriate for the data because the appropriate model is needed to get valid evidence for policymakers. The use of a weighted nationally representative EDHS data and the use of advanced survival model (Gompertz gamma shared frailty model) were therefore the strengths of the study. On the other hand, the limitations of this study could be that the EDHS survey did not incorporate the communities’ and respondents’ knowledge, attitude, and perception towards PNC service utilization, such as the community’s level of awareness, norms, and beliefs towards PNC. This study also lacks the assessment of the capacity and accessibility of the local health systems, the level of decentralized decision making, and common cultural practices, particularly cultural practices regarding seclusion that may reduce care-seeking. Moreover, the data were collected based on the mother’s or caregiver’s report and might introduce the possibility of social desirability and recall bias.

## Conclusion

The study indicated that early initiation of postnatal care utilization was low in Ethiopia. A very low proportion of women received postnatal care within the most critical period (within 24 h) after delivery. It is important to ascertain further why postnatal care rates are so low. Though barriers to receiving postnatal care were antenatal care, media exposure, place of delivery, mode of delivery, and access to maternal health services were important predictors for the utilization of early postnatal care. The policymakers and implementers should promote the utilization of antenatal care and institutional delivery service utilization through mass media. The government should also design a new approach to enhance the uptake of postnatal care services for poor households and to scale up the PNC services, including the different possibilities for women who give births at the health facilities and homes. Future researchers had better assess the capacity and accessibility of the local health systems, the level of decentralized decision making, common cultural practices, knowledge, attitude, and perception of mothers towards PNC service utilization.

## Supplementary Information

**Additional file 1.** Proportional hazard assumption for the incidence of early initiation of PNC utilization and its predictors among women who gave births in the last 2 years in Ethiopia, 2016

**Additional file 2.** Log rank test for the predictors of PNC services utilizations among mothers who gave birth in the last 2 years, 2016

**Additional file 3.** Model comparison and diagnostics for PNC service utilization among mothers in Ethiopia, 2016

## Data Availability

The datasets used during the current study are available at Measure DHS website: http://www.measuredhs.com.

## Abbreviations

ANC: Antenatal Care
EA: Enumeration Area
EDHS: Ethiopia Demography Health Survey
PNC: Post Natal Care
SD: Standard Deviation
USD: US Dollar
WHO: World Health Organization
