# Supplementary material for: Time to early initiation of postnatal care service utilization and its predictors among women who gave births in the last 2 years in Ethiopia: a shared frailty model
Source: Arch Public Health. 2021 Apr 15;79:51. doi: 10.1186/s13690-021-00575-7 (PMC8048056; doi:10.1186/s13690-021-00575-7)
Supplement: Supplementary file 1 — Additional file 1. Proportional hazard assumption for the incidence of early initiation of PNC utilization and its predictors among women who gave births in the last 2 years in Ethiopia, 2016 [file 13690_2021_575_MOESM1_ESM.docx]

**Table S1**: Proportional hazard assumption for the incidence of early initiation of PNC utilization and its predictors among women who gave births in the last 2 years in Ethiopia, 2016

| **Variables** | **Rho** | **Chi2** | **Df** | **Prob>chi2** |
| --- | --- | --- | --- | --- |
| Residence | 0.007 | 0.10 | 1 | 0.75 |
| Wealth index | -0.044 | 3.60 | 1 | 0.058 |
| Twin | 0.011 | 0.20 | 1 | 0.66 |
| ANC | 0.071 | 7.86 | 1 | 0.006 |
| Media exposure | -0.015 | 0.39 | 1 | 0.53 |
| Parity | -0.022 | 0.91 | 1 | 0.34 |
| Maternal education | 0.0009 | <0.0001 | 1 | 0.97 |
| Husband education | -0.022 | 0.91 | 1 | 0.34 |
| Health care access problem | 0.063 | 8.61 | 1 | 0.003 |
| Women autonomy | 0.019 | 0.66 | 1 | 0.42 |
| Preceding birth interval | 0.003 | 0.02 | 1 | 0.88 |
| Marital status | -0.006 | 0.09 | 1 | 0.76 |
| Maternal age | -0.016 | 0.54 | 1 | 0.46 |
| Sex of household head | -0.011 | 0.25 | 1 | 0.61 |
| Place of delivery | -0.28 | 127.77 | 1 | <0.0001 |
| Mode of delivery | 0.049 | 5.39 | 1 | 0.02 |
| Birth outcome | 0.067 | 9.65 | 1 | 0.0009 |
| Global test |  | 194.52 | 17 | <0.0001 |
