# Supplementary material for: Time to early initiation of postnatal care service utilization and its predictors among women who gave births in the last 2 years in Ethiopia: a shared frailty model
Source: Arch Public Health. 2021 Apr 15;79:51. doi: 10.1186/s13690-021-00575-7 (PMC8048056; doi:10.1186/s13690-021-00575-7)
Supplement: Supplementary file 2 — Additional file 2. Log rank test for the predictors of PNC services utilizations among mothers who gave birth in the last 2 years, 2016 [file 13690_2021_575_MOESM2_ESM.docx]

**Table S2**: Log rank test for the predictors of PNC services utilizations among mothers who gave birth in the last 2 years, 2016

| Variables | p-value | Variables | p-value |
| --- | --- | --- | --- |
| Residence | <0.0001 | Parity | <0.0001 |
| Region | <0.0001 | Media exposure | <0.0001 |
| Sex of household head | <0.0001 | Maternal education | < 0.0001 |
| Wealth index | <0.0001 | Husband education | <0.0001 |
| Birth outcome | 0.243 | Mode of delivery | <0.0001 |
| Health care access problem | <0001 | Women decision making autonomy | <0.0001 |
| Place of delivery | <0.0001 | Maternal age | <0.0001 |
| ANC visit during pregnancy | <0.0001 | Number of births | <0.0001 |
| Birth interval | <0.0001 | Marital status | 0.033 |
