# Supplementary material for: Time to early initiation of postnatal care service utilization and its predictors among women who gave births in the last 2 years in Ethiopia: a shared frailty model
Source: Arch Public Health. 2021 Apr 15;79:51. doi: 10.1186/s13690-021-00575-7 (PMC8048056; doi:10.1186/s13690-021-00575-7)
Supplement: Supplementary file 3 — Additional file 3. Model comparison and diagnostics for PNC service utilization among mothers in Ethiopia, 2016 [file 13690_2021_575_MOESM3_ESM.docx]

Table S3: Model comparison and diagnostics for PNC service utilization among mothers in Ethiopia, 2016

| **Model** | **Distribution** | **Frailty** | **Theta** | **AIC** | **BIC** | **Deviance (-2LL)** |
| --- | --- | --- | --- | --- | --- | --- |
| Shared frailty | Gompertz | Gamma | 0.24 | 6277.04 | 6429.74 | 6231.04 |
| Shared frailty | Gompertz | Inverse Gaussian | 0.30 | 6273.01 | 6425.71 | 6227.01 |
| Shared frailty | Exponential | Gamma | 0.24 | 6277.76 | 6423.82 | 6233.76 |
| Shared frailty | Exponential | Inverse Gaussian | 0.29 | 6273.4 | 6419.91 | 6229.84 |
| Shared frailty | Weibull | Gamma | 0.16 | 5968.55 | 6121.25 | 5924.14 |
| Shared frailty | Weibull | Inverse Gaussian | 0.18 | 5970.15 | 6122.85 | 5922.54 |
